# Highly Selenite-Tolerant Strain *Proteus mirabilis* QZB-2 Rapidly Reduces Selenite to Selenium Nanoparticles in the Cell Membrane

**DOI:** 10.3389/fmicb.2022.862130

**Published:** 2022-04-11

**Authors:** JinLan Huang, DaiHua Jiang, MingShi Wang, XueJiao Huang

**Affiliations:** Guangxi Colleges and Universities Key Laboratory of Crop Cultivation and Tillage, Guangxi University, Nanning, China

**Keywords:** *Proteus mirabilis*, selenite reduction, membrane, selenium nanoparticles, reduction efficiency

## Abstract

The application of biosynthesized nano-selenium fertilizers to crops can improve their nutrient levels by increasing their selenium content. However, microorganisms with a high selenite tolerance and rapid reduction rate accompanied with the production of selenium nanoparticles (SeNPs) at the same time have seldom been reported. In this study, a bacterial strain showing high selenite resistance (up to 300 mM) was isolated from a lateritic red soil and identified as *Proteus mirabilis* QZB-2. This strain reduced nearly 100% of 1.0 and 2.0 mM selenite within 12 and 18 h, respectively, to produce SeNPs. QZB-2 isolate reduced SeO_3_^2^*^–^* to Se^0^ in the cell membrane with NADPH or NADH as electron donors. Se^0^ was then released outside of the cell, where it formed spherical SeNPs with an average hydrodynamic diameter of 152.0 ± 10.2 nm. *P. mirabilis* QZB-2 could be used for SeNPs synthesis owing to its simultaneously high SeO_3_^2^*^–^* tolerance and rapid reduction rate.

## Highlights

- Microorganisms with a high selenite tolerance, selenite reduction capacity, and also the ability to produce selenium nanoparticles (SeNPs) have rarely been reported.
- Here, we report a *Proteus mirabilis* QZB-2 strain with a high selenite resistance (up to 300 mM).
- This strain exhibited excellent SeO_3_^2^*^–^* reduction efficiency, nearly exhausted all of the SeO_3_^2^*^–^* (1 or 2 mM) within 18 h and transformed SeO_3_^2^*^–^* into SeNPs more rapidly than any other bacteria reported to date.
- The SeO_3_^2^*^–^* reduction activity in QZB-2 cells was attributed to the cell membrane fraction, with or without NADH/NADPH serving as electron donors.
- Our results reveal that *P. mirabilis* QZB-2 is an attractive bacterial candidate for the synthesis of novel nano-Se fertilizers owing to its simultaneously high SeO_3_^2^*^–^* tolerance and rapid reduction rate.

## Introduction

Selenium (Se) is an essential trace nutrient for humans and plays an important role in maintaining body health and preventing diseases (Dinh et al., 2018; Yan et al., 2021). However, globally, 0.5–1 billion people suffer from Se deficiency (Li et al., 2021). The biofortification of crops with Se-rich fertilizers is considered to be the most effective way to increase human Se intake (Luo et al., 2019; Zhang et al., 2019a; Li et al., 2021). While fertilizers can contain organic selenium and nano-selenium, selenite (SeO_3_^2^*^–^*) and selenate (SeO_4_^2^*^–^*) are the two major types of inorganic Se used in fertilizers because of their low cost. However, the utilization efficiency of selenite and selenate by plants is relatively low because of their high toxicity (Deng et al., 2017). Soluble nano-Se is less toxic than inorganic and organic Se and has a higher bioavailability (Zahedi et al., 2019; Kumar and Prasad, 2021). The application of nano-Se to foliage or soil can significantly enhance crop yield, quality, and Se content (Li et al., 2020, 2021). Thus, improving the processes for synthesizing of nano-Se fertilizers could enhance the development of Se-rich agricultural products.

Traditional physical and chemical methods to synthesize selenium nanoparticles (SeNPs) are costly and cause pollution. Therefore, biological approaches are generally preferred because they have a low-cost and are eco-friendly (Wang et al., 2018b; Kumar and Prasad, 2021). Studies have revealed that some bacterial strains, such as *Pseudomonas* spp. (Kuroda et al., 2011; Avendaño et al., 2016), *Bacillus* spp. (Blum et al., 1998; Oremland et al., 2004; Bao et al., 2016), *Clostridium* sp. (Bao et al., 2013), *Selenihalanaerobacter* sp. (Oremland et al., 2004), and *Sulfurospirillum* sp. (Oremland et al., 2004), can synthesize nano-Se. Most of them use SeO_3_^2^*^–^* as a raw material, but only a few can use both SeO_3_^2^*^–^* and SeO_4_^2^*^–^*. Moreover, most Se-reducing microorganisms have a limited tolerance to SeO_3_^2^*^–^* (≤100 mM) and require 48 h or more to reduce all SeO_3_^2^*^–^* (≥1 mM) to elemental selenium (Se^0^) (Table 1). Therefore, novel strains with high SeO_3_^2^*^–^* tolerance and robust SeO_3_^2^*^–^* reduction abilities are required to improve SeNP synthesis.

**TABLE 1:** Some bacteria for reduction of selenium.

Bacteria	Tolerance of selenium		Reduction ability			References
		
	Se(IV)	Se(VI)	Starting selenium	Time	Reduction rate	
*Stenotrophomonas maltophilia*	—	—	0.5 mM Se(VI), 0.5 mM Se(IV)	48 h	99.8%, 81.2%	Dungan et al., 2003
*Citrobacter braakii*	—	—	2.3–3.2 μg/L Se(VI)	192 h	87–97%	Zhang and Frankenberger, 2006
*Bacillus* sp. RS1	—	—	1 mg/L Se(VI)	192 h	57%	Zhang and Frankenberger, 2007
*Bacillus* sp. STG-83	640 mM	320 mM	1 mM Se(VI), 1 mM Se(IV)	96 h	100%, 100%	Soudi et al., 2008
*Pseudomonas stutzeri* NT-I	94 mM	122 mM	0.9 mM Se(IV)	18 h	100%	Kuroda et al., 2011
*Clostridium* sp. BXM	—	—	1 mM Se(VI), 1 mM Se(IV)	360 h	36–49%	Bao et al., 2013
*Rhodopseudomonas palustris* N	8 mM	—	2 mM Se(IV)	192 h	82.00%	Li et al., 2014a
*Comamonas testosteroni* S44	100 mM	—	1 mM Se(IV)	24 h	40%	Zheng et al., 2014
*Shewanella oneidensis* MR-1	—	—	0.5 mM Se(IV)	12 h	82%	Li et al., 2014b
*Bacillus mycoides* SeiTE01	—	—	2 mM Se(IV)	24 h	100%	Lampis et al., 2014
*Pseudomonas putida KT2440*	10 mM	—	1 mM Se(IV)	24 h	89%	Avendaño et al., 2016
*Bacillus oryziterrae ZYK*	—	—	1 mM Se(IV)	360	90%	Bao et al., 2016
*Stenotrophomonas maltophilia* SeITE02	—	—	2 mM Se(IV)	192 h	86%	Lampis et al., 2017
*Enterobacter cloacae* Z0206	—	—	2 mM Se(IV)	72 h	100%	Song et al., 2017
*Stenotrophomonas bentonitica* BII-R7	200 mM	—	2 mM Se(IV)	48 h	100%	Ruiz Fresneda et al., 2018
*Rahnella aquatilis* HX2	85 mM	590 mM	10 mM Se(VI), 5 mM Se(IV)	48 h	38.5%, 39.8%	Zhu et al., 2018
*Alcaligenes faecalis* Se03	120 mM	—	5 mM Se(IV)	48 h	100%	Wang et al., 2018a
*Proteus mirabilis* YC801	100 mM	—	1 mM Se(IV)	42 h	100%	Wang et al., 2018b
*Bacillus safensis* JG-B5T	—	—	2.5 mM Se(IV)	336 h	70%	Fischer et al., 2019
*Providencia rettgeri* HF16-A	100 mM	—	1 mM Se(IV)	42 h	100%	Huang et al., 2021

In this study, the aerobic bacterium *P. mirabilis* QZB-2, isolated from lateritic red soil in Guangxi, China, showed strong tolerance to high concentrations of SeO_3_^2^*^–^* (up to 300 mM). The QZB-2 strain reduced the majority of 1.0 and 2.0 mM SeO_3_^2^*^–^* to Se^0^ within 12 and 18 h, respectively. This reduction occurred in the cell membrane, after which Se^0^ was released outside the cell where it formed spherical SeNPs with an average hydrodynamic diameter of 152.0 ± 10.2 nm. Our results reveal that *P. mirabilis* QZB-2 is a strong bacterial candidate for the synthesis of novel nano-Se fertilizers owing to its simultaneously high SeO_3_^2^*^–^* tolerance and rapid reduction rates.

## Materials and Methods

### Culture Medium

Luria–Bertani (LB) medium (per liter, pH 7.0–7.2) was used for bacterial enrichment. It contained 5.00 g of yeast extract, 10.00 g of NaCl, and 10.00 g of tryptone per liter. A Na_2_SeO_3_ solution was prepared in deionized water and sterilized by filtration.

### Isolation and Identification of Selenite-Reducing Bacteria

Soil samples were collected from a naturally occurring Se-rich lateritic red soil (0–15 cm depth) on dry land in Guangxi province, southern China (22°05′31′′ N, 108°32′53′′ E). The total Se in the soil was 0.53 mg/kg. To isolate Se-reducing bacteria, 1 g of the soil sample was suspended in 100 mL of sterilized LB broth supplemented with 1 mM SeO_3_^2^*^–^*, and cultured at 30°C (150 rpm) for 48 h. The bacterial strains were subcultured three times with an inoculum size of 5%. The culture solution was then diluted three times (from 10^–5^ to 10^–7^), and 100 μL of each dilution was inoculated on LB agar plates containing 10.00 mM SeO_3_^2^*^–^*. The agar plates were incubated at 30°C for 24 h. Red colonies were continuously picked and subcultured onto new plate until the pure cultures were finally obtained. Of all the monocultures, the QZB-2 isolate was chosen for further experiments because of its high SeO_3_^2^ tolerance.

To identify the QZB-2 isolate, its cell morphology was determined using an Olympus BH-2 optical microscope. The antibiotic resistance of the QZB-2 strain was tested using 1 and 100 μg/mL tetracycline, ampicillin, chloramphenicol, kanamycin, and gentamycin. Subsequently, the 16S rRNA gene was amplified using universal primers 27F (5′-AGAGTTTGATCMTGGCTCAG-3′) and 1492R (5′-ACGGTTACCT TGTTACGACTT-3′) (Zhang et al., 2019b). The obtained sequence was compared with other previously published sequences of the bacterial 16S rRNA gene in the NCBI. Finally, a phylogenetic tree was constructed using MEGA 7.0 software (Huang et al., 2018b).

### Tolerance and Reduction of Selenite by the QZB-2 Strain

#### Selenite Tolerance

The tolerance of the QZB-2 isolate to SeO_3_^2^*^–^* was assessed by determining the minimal inhibitory concentration (MIC) of SeO_3_^2^*^–^* (Huang et al., 2021). First, the QZB-2 strain was activated in an LB medium and then harvested at 3,500 × *g* for 5 min. The harvested cells were washed with a phosphate buffer (PBS) and inoculated into fresh LB medium supplemented with different concentrations of SeO_3_^2^*^–^* (0–600 mM). The culture was incubated at 30°C and 150 rpm for 24 h. Subsequently, 100 μL of the culture cells was inoculated onto LB agar plates and incubated for an additional 72 h at 30°C to determine the SeO_3_^2^*^–^* concentration that inhibited the growth of the QZB-2 strain.

#### Selenite Reduction

The activated QZB-2 strain was inoculated into an LB medium containing 1 or 2 mM SeO_3_^2^*^–^*. Two types of negative controls, without SeO_3_^2^*^–^* or without bacteria, were used. The cultures were incubated at 30°C on a shaker (150 rpm) for 36 h. The SeO_3_^2^*^–^* and Se^0^ contents, as well as the amount of bacterial growth, were determined every 6 h.

#### Analytical Methods

Bacterial growth was determined based on the number of colony-forming units (CFUs), which was determined by spreading 100 μL of the diluted culture on LB plates and incubating them at 30°C for 72 h. SeO_3_^2^*^–^* concentrations were determined using an atomic fluorescence morphology analyzer (SA-20; Jitian, Beijing), while Se^0^ content was measured using a spectrophotometric method, as described by Khoei et al. (2017).

### Subcellular Localization of Selenite Reduction

Different fractions of QZB-2 were collected to determine the cell compartment where SeO_3_^2^*^–^* is reduced. First, QZB-2 isolate was incubated in an LB medium for 18 h (stationary phase) and centrifuged at 10,000 × *g* for 10 min at 4°C. The obtained bacterial cell pellets were washed twice with 0.9% NaCl and then treated with different reagents to extract the periplasmic, membrane, and cytoplasmic fractions, as reported by Khoei et al. (2017). Pellets were treated with lysozyme and EDTA for 10 min, then centrifuged at 20,000 × *g* for 30 min, after which the supernatant was collected as a periplasmic fraction. Subsequently, the pellets were resuspended in 50 mM NaCl and then disrupted by ultra-sonication for 15 min. The supernatant was harvested as a cytoplasmic fraction following the centrifugation of the suspension at 20,000 × *g* for 70 min. The membrane fraction was obtained after the pellet were resuspended in 50 mM phosphate-buffered saline (PBS) (pH 7.4).

To extract extracellular polymeric substance (EPS), QZB-2 isolate was incubated in an LB medium at 30°C for 5 day. Following this, the cultures were centrifuged at 10,000 × *g* for 30 min at 4°C to obtain the supernatant. After passing the supernatant through a 0.45 μm filter, the supernatant was mixed with pre-cooled ethanol (1:1) and precipitated at −20°C overnight. Finally, precipitated EPS was collected by centrifugation (10,000 × *g*, 30 min, 4°C) (Wang et al., 2018b). For supernatant preparation, the stationary phase cultures (18 h) were centrifuged at 10,000 × *g* for 10 min and 4°C, before the supernatant was passed through a 0.22 μm filter and collected (Khoei et al., 2017).

Activity assays were conducted on a 96-well plate, where 100 μL of intracellular and extracellular fractions were briefly added to each well that contained 88 μL of PBS, 10 μL of SeO_3_^2^*^–^* solution (2.0 mM), and 2 μL of NADH or NADPH (electron donor, 2.0 mM). The plate was then cultivated at 30°C for 72 h. Plates without an electron donor, a cell fraction (the supernatant/cell protein/EPS), or SeO_3_^2^*^–^* were used as negative controls.

### Localization and Characterization of Selenium

QZB-2 isolate was cultured in an LB medium, either supplemented with 2 mM SeO_3_^2^*^–^* or without SeO_3_^2^*^–^*. After 24 h, the culture was centrifuged (5,000 × *g* for 10 min) to collect bacterial cell pellets. For transmission electron microscopy (TEM) analysis, the pellet was fixed with 2.5% glutaraldehyde at 4°C for 24 h and dried in an ultra-low temperature freezer (ALPHAL-4LD PLUS, CHRIST Co., Germany) (Huang et al., 2021). For scanning electron microscopy-energy dispersive X-ray spectrometry (SEM-EDS), the pellet was fixed with 2.5% glutaraldehyde at 4°C for 24 h and then dehydrated in ethanol solutions (30, 50, 70, 90, and 100%) before drying.

### Analysis of Selenium Nanoparticles

First, the QZB-2 isolate was incubated in an LB medium containing 2 mM SeO_3_^2^*^–^* for 24 h and centrifuged at 10,000 × *g* for 10 min at 4°C. The resulting bacterial cell pellets were washed twice with 0.9% NaCl, resuspended in Tris-Cl buffer, and then disrupted by ultra-sonication. The SeNPs were harvested after the cell suspension was centrifuged at 40,000 × *g* for 40 min at 4°C. Dynamic Light Scattering (DLS) and zeta potential analysis of SeNPs was conducted using a Nano-ZS90X Zeta potential particle size tester (Malvern, Britain) (Lampis et al., 2017). Afterward, the purified SeNPs were dried in an ultra-low temperature freezer (ALPHAL-4LD PLUS, CHRIST Co., Germany) for SEM-EDS, XRD, and FTIR analysis. Furthermore, the morphology and constituent elements analysis of SeNPs were analyzed using SEM-EDS. The compounds of SeNPs were analyzed using a D/Max-3C X-ray diffractometer with Cu-K radiation in the range of 10–80° (2θ) at a scan rate of 2°/min. The possible chemical bonds in SeNPs were investigated using a Fourier transformed infrared (FTIR) spectrophotometer in the range of 4,000–400 cm^–1^ (Huang et al., 2018a).

### Statistical Analyses

The obtained data were analyzed by one-way analysis of variance (ANOVA) using SPSS Statistics 22 software. The level of statistical significance was set at *p* < 0.05. Graphics were plotted using Origin 8.6.

## Results and Discussion

### Characterization and Identification of the QZB-2 Strain

**FIGURE 1:**
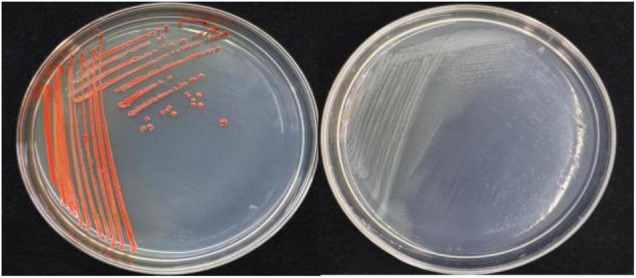
Growth of strain QZB-2 on LB agar plates (left: supplied with 2 mM SeO_3_^2–^; left: no SeO_3_^2–^). The red colony color indicates selenite reduction and the formation of elemental selenium.

**FIGURE 2:**
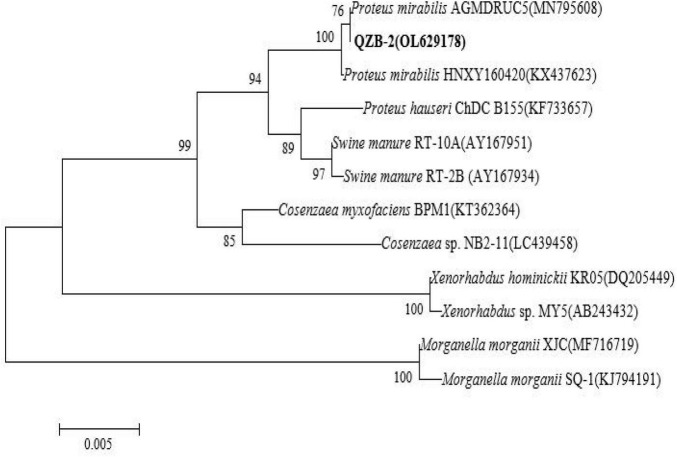
Maximum likelihood tree based on the 16S rRNA gene sequence of isolate QZB-2. The scale bars indicate 0.005 substitutions per site.

**FIGURE 3:**
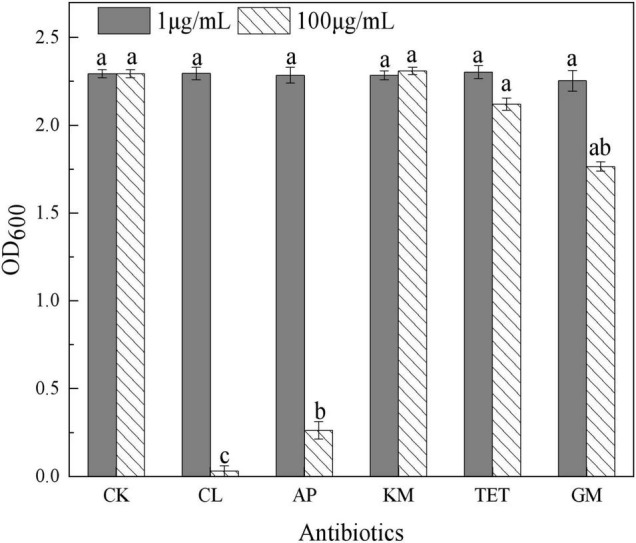
Influence of antibiotics on *Proteus mirabilis* QZB-2 growth in 24 h (CK: no antibiotics; CL: chloramphenicol; AP: ampicillin; KM: kanamycin; TET: 2.212e; GM: gentamycin). Different letters indicate sinificant differences between treatments at *P* < 0.05.

In this study, 20 bacterial strains were isolated from naturally occurring Se-rich lateritic red soil in Guangxi, China, using LB plates supplemented with 20 mM SeO_3_^2^*^–^*. Of these strains, the QZB-2 isolate was chosen for further experiments as it exhibited good growth and high SeO_3_^2^*^–^* reduction ability (Figure 1). Phylogenetic analysis indicated that the QZB-2 isolate was closely related to *P. mirabilis* AGMDRUC5 (MN795608) (Figure 2) and was therefore identified as *P. mirabilis* QZB-2 (OL629178). *Proteus* sp. is a rod-shaped gram-negative bacterium that is commonly found in the environment (Drzewiecka, 2016), with some strains exhibiting heavy metal (Cr^6+^, Cu^2+^, Zn^2+^) resistance (Rani et al., 2008; Ge et al., 2013; Islam et al., 2014). Recently, Wang et al. (2018a) reported that *P. mirabilis* YC801 isolated from insect guts could tolerate 100 mM SeO_3_^2^*^–^*. In this study, MIC assays showed that *P. mirabilis* QZB-2 could grow and produce Se^0^ even at a SeO_3_^2^*^–^* concentration of 300 mM (Figure 1). Moreover, *P. mirabilis* QZB-2 isolated from soil was resistant to several common antibiotics at concentrations from 1 to even 100 μg/mL (Liu et al., 2021; Figure 3). Thus, we further explored the characteristics of SeO_3_^2^*^–^* reduction and SeNPs production in the QZB-2 strain.

### Selenite Reduction Characteristics by the QZB-2 Strain

**FIGURE 4:**
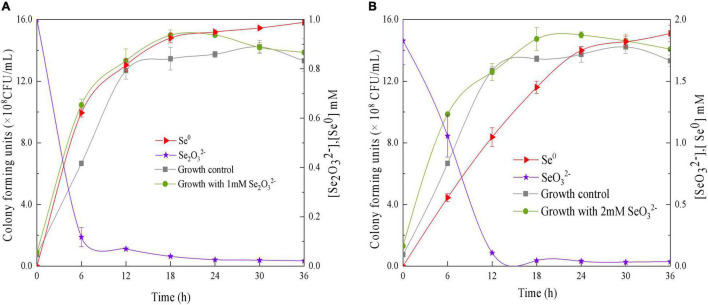
Time courses of bacterial growth, SeO_3_^2–^ removal, and Se^0^ formation by the strain *Proteus mirabilis* QZB-2 grown in LB medium containing 1 mM **(A)** and 2 mM **(B)** SeO_3_^2–^.

The ability of QZB-2 to reduce SeO_3_^2^*^–^* was studied in liquid LB medium containing 1.0 or 2.0 mM of SeO_3_^2^*^–^*. Our results show that the relative growth curves of QZB-2 in a SeO_3_^2^*^–^* containing culture (both 1 and 2 mM) followed the same pattern as that in cultures without SeO_3_^2^*^–^* (Figure 4). These patterns confirmed that 1.0 and 2.0 mM of SeO_3_^2^*^–^* did not inhibit QZB-2 growth. SeO_3_^2^*^–^* depletion occurred at the start of the growth phase in the culture with 1.0 mM SeO_3_^2^*^–^* (Figure 4A). These findings are similar to those reported by Wang et al. (2018a), who found that SeO_3_^2^*^–^* was reduced by *P. mirabilis* YC801 after 6 h of incubation. However, *P. mirabilis* YC801 only reduced approximately 15% of the total SeO_3_^2^*^–^*, while the QZB-2 strain reduced the majority of the SeO_3_^2^*^–^* (88.0%) in 6 h (Figure 4). In most Se-reducing microorganisms, SeO_3_^2^*^–^* reduction occurs at the beginning or during the mid-exponential phase (≥12 h) (Avendaño et al., 2016; Lampis et al., 2017; Ruiz Fresneda et al., 2018). However, these microorganisms require more time (usually over 24 h) to reduce the majority of the SeO_3_^2^*^–^* (1 or 2 mM). In this study, the QZB-2 strain reduced both 1 and 2 mM of SeO_3_^2^*^–^* at the beginning of its growth and nearly exhausted all of the SeO_3_^2^*^–^* within 18 h. These results indicate that *P. mirabilis* QZB-2 has an excellent SeO_3_^2^*^–^* reduction efficiency.

In the QZB-2 strain, SeO_3_^2^*^–^* reduction was coupled with Se^0^ accumulation; after 6 h, 88% of the 1 mM SeO_3_^2^*^–^* was depleted while 70.45% of which was reduced to Se^0^ (Figure 4A). Conversely, a delay in Se^0^ formation has been observed in some bacteria, including *B. mycoides* SeITE01 (Lampis et al., 2014), *S. maltophilia* SeITE02 (Lampis et al., 2017), *A. faecalis S*e03 (Wang et al., 2018b), and *P. mirabilis* YC801 (Tugarova et al., 2018). We observed that in the QZB-2 strain more than 90% of the reduced SeO_3_^2^*^–^* (1 and 2 mM) was transformed into Se^0^ after 36 h of incubation (Figure 4). These results demonstrate that *P. mirabilis* QZB-2 can tolerate high concentrations of SeO_3_^2^*^–^* and transforms SeO_3_^2^*^–^* into SeNPs more rapidly than any other bacteria reported to date.

### Subcellular Localization of Selenite Reduction

**FIGURE 5:**
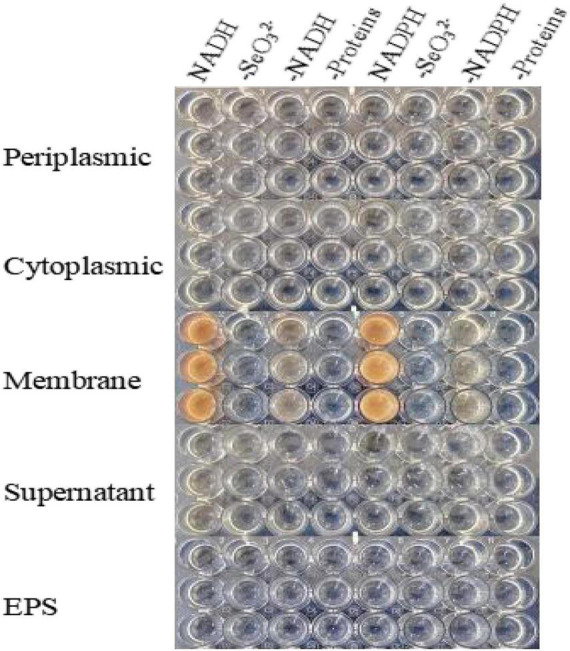
*In vitro* SeO_3_^2^*^–^* reducing activity assays on different subcellular fractions (cytoplasmic, periplasmic, and membrane), supernatant, and exopolysaccharide (EPS). All experiments were performed in duplicate, with addition of 2 mM SeO_3_^2–^ and 2 mM NADPH or NADH. While 3 following control negatives were performed: without protein fractions, without selenite, without NADPH or NADH.

**FIGURE 6:**
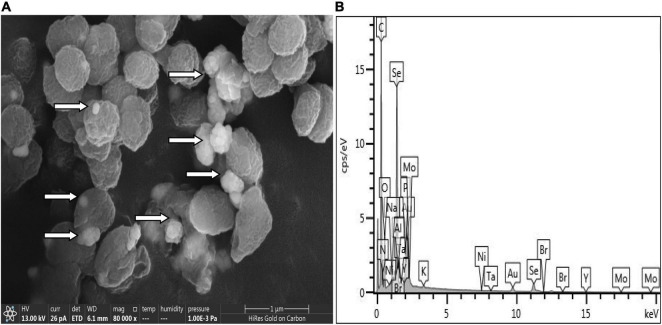
Scanning electron microscopy (SEM) **(A)** and EDX **(B)** analysis of *Proteus mirabilis* QZB-2 cultures grown in presence of 2 mM selenite.

**FIGURE 7:**
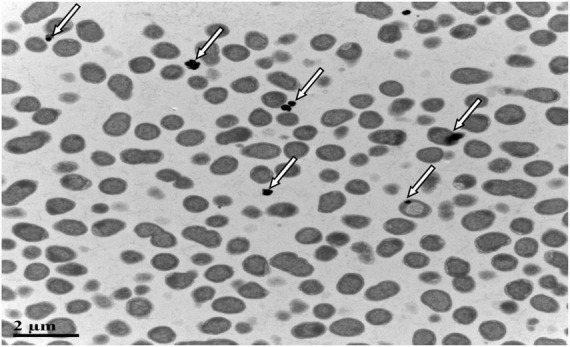
Transmission electron microscopy (TEM) micrographs showing SeNPs produced by *Proteus mirabilis* QZB-2 after 24 h of incubation with 2 mM sodium selenite.

SeO_3_^2^*^–^* reduction activity in the QZB-2 strain was localized in the cell membrane fraction (Figure 5), whereas in *A. faecalis* Se03, *P. mirabilis* YC801, and *P. rettgeri* HF16 cells, reduction activity is localized in the cytoplasmic fraction (Wang et al., 2018a,b; Huang et al., 2021). This shows that the SeO_3_^2^*^–^* reduction mechanism in QZB-2 is inconsistent with that observed in earlier studies. SeO_3_^2^*^–^* reduction in the QZB-2 strain only occurred when NADH or NADPH was present (Figure 5), which is consistent with reported SeO_3_^2^*^–^* reduction in *S. maltophilia* SeITE02 (Lampis et al., 2017) and *B. fungorum* strains (Khoei et al., 2017). Thus, we propose that SeO_3_^2^*^–^* reduction by *P. mirabilis* QZB-2 occurred in the cell membrane system through the catalytic activity of a reductase, with NADH/NADPH serving as electron donors. Moreover, SEM and EDX analysis show that SeNPs were found on the QZB-2 cell surface (Figures 6A,B), while TEM analysis illustrates that SeNPs were located in the extracellular space or cell membrane (Figure 7). Altogether, these findings suggest that the QZB-2 strain produced SeNPs within the cell and then released them into the medium.

### Characterization of Selenium Nanoparticles

**FIGURE 8:**
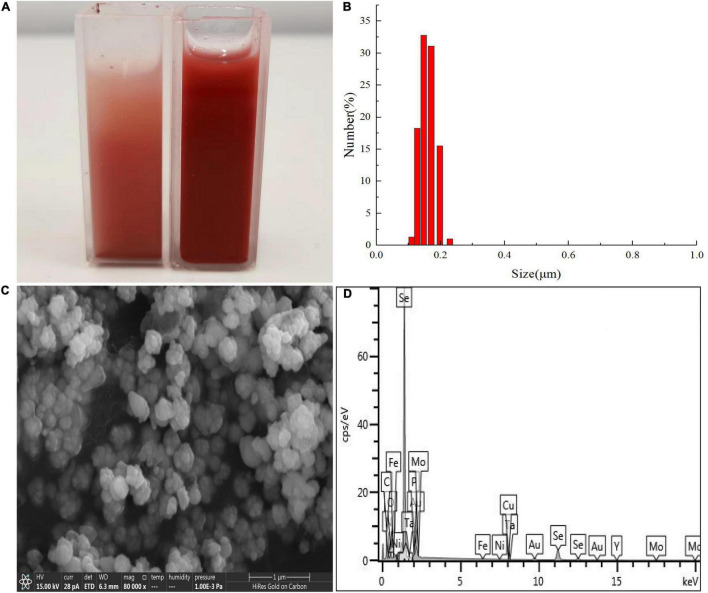
Dynamic light scattering (DLS) spectra of purified SeNPs produced by *Proteus mirabilis* QZB-2 in LB supplemented with 2.0 mM selenite. **(A)** Selenite-dosing cells (left) and purified nano-selenium (right); **(B)** size distribution of purified SeNPs; **(C)** SEM micrographs of purified SeNPs. **(D)** EDX analysis of purified SeNPs showing its selenium composition.

**FIGURE 9:**
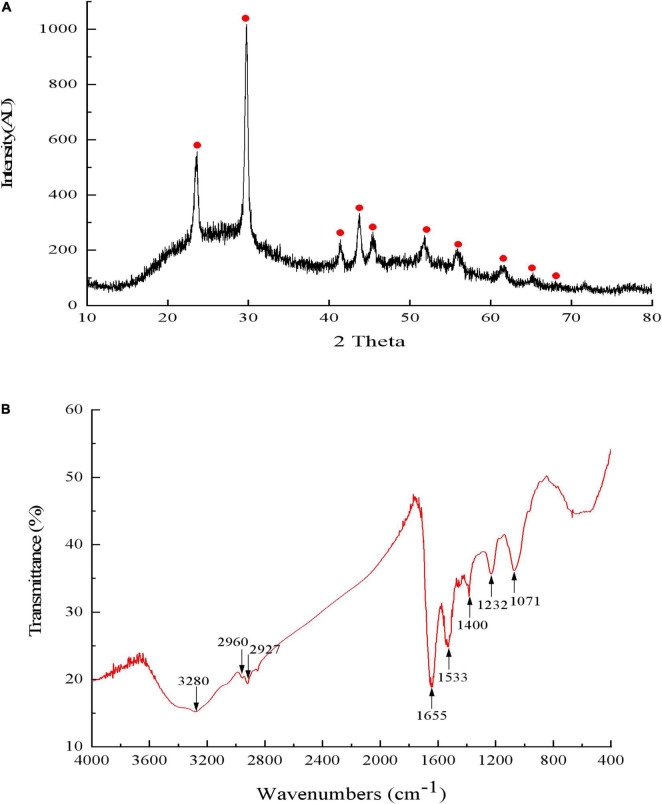
The X-ray diffraction spectrogram **(A)** and FTIR spectrum **(B)** of SeNPs synthesized by *Proteus mirabilis* QZB-2.

Results of DLS analysis of the purified SeNPs are presented in Figures 8A,B. They had an average hydrodynamic diameter of 152.0 ± 10.2 nm (Figures 8B,C). Moreover, the existence of Se was determined by EDX analysis, with selenium-specific peaks observed at 1.38 and 11.22 keV (Figure 8D), and Figure 9A shows the XRD traces of SeNPs. The diffraction peaks at 2θ = 23.6, 29.7, 41.3, 43.7, 45.4, 51.9, 56.3, 61.9, 65.2, and 68.6° indicated the presence of the pure selenium in the sample (Shin et al., 2007). In addition, the FTIR spectra of the SeNPs are shown in Figure 9B. The absorption band at 3,280 cm^–1^ is due to the stretching vibration of protein-modified N-H, amide A, or amide II (Zonaro et al., 2017; Wang et al., 2018b). The weak absorption peaks at 2,960 and 2,927 cm^–1^ represent the symmetric stretching vibrations of C-H in the sugar ring or peptide chain (Xu et al., 2018). The peaks at 1655, 1533, and 1232 cm^–1^ are accompanied by a low-intensity band and represent amide I, amide II, and amide III, respectively, which is a typical protein pattern (Kamnev et al., 2017; Wang et al., 2018b). The peak at 1,400 cm^–1^ is because of the symmetric stretching vibration of carboxylate (COO−), while its asymmetric counterpart can be seen at 1,655 cm^–1^ (Lampis et al., 2017; Zonaro et al., 2017; Wang et al., 2018b). The bands at 1,071 cm^–1^ are typical of C–O vibrations in carbohydrates, which may suggest the existence of polysaccharides (Tugarova et al., 2018; Wang et al., 2018b). The results of FTIR analysis clearly show that the surface of the SeNPs produced by *P. mirabilis* QZB-2 contained organic residues from carbohydrates, lipids, and proteins. The composition of these organic groups was in accordance with those produced by *S. maltophilia* SeITE02 and *P. mirabilis* YC801 (Lampis et al., 2017; Wang et al., 2018a). These organic groups could participate in SeO_3_^2^*^–^* reduction, as well as SeNP formation and stabilization processes.

A previous study showed that selenite and selenate were not suitable additives when applied as plant growth fertilizers owing to their high toxicity (Deng et al., 2017). However, when using as a fertilizer, nano-Se could significantly enhance the Se content of crops (Li et al., 2020, 2021), which can safely increase human Se intake. Our study found that strain QZB-2 could tolerate high level of SeO_3_^2^*^–^*; it rapidly reduces SeO_3_^2^*^–^* to Se^0^ while simultaneously synthesizing SeNPs. This result indicates strain QZB-2 could be used for SeNPs synthesis. However, the application of these synthesized nanoparticles in fertilizer has not been well-characterized and requires further study.

## Conclusion

The highly selenite-tolerant (up to 300 mM) strain *P. mirabilis* QZB-2 was isolated from a naturally occurring Se-rich paddy soil. QZB-2 reduced almost all selenite to form selenium nanoparticles within 18 h. The SeO_3_^2^*^–^* reduction activity in QZB-2 cells was attributed to the cell membrane fraction with NADH/NADPH serving as electron donors. Therefore, the QZB-2 bacterial strain is a promising candidate for the production of novel nano-selenium fertilizers owing to its simultaneously high SeO_3_^2^*^–^* tolerance and rapid reduction rate.

## Data Availability Statement

The original contributions presented in the study are publicly available. This data can be found here: OL629178.

## Author Contributions

XH and DJ: conceptualization, writing—review and editing, and funding acquisition. JH: methodology, data curation, visualization, supervision, and writing—original draft preparation. MW: software, formal analysis, and investigation. XH, MW, and DJ: validation. XH: resources and project administration. All authors have read and agreed to the published version of the manuscript.

## Conflict of Interest

The authors declare that the research was conducted in the absence of any commercial or financial relationships that could be construed as a potential conflict of interest.

## Publisher’s Note

All claims expressed in this article are solely those of the authors and do not necessarily represent those of their affiliated organizations, or those of the publisher, the editors and the reviewers. Any product that may be evaluated in this article, or claim that may be made by its manufacturer, is not guaranteed or endorsed by the publisher.
