# Supplementary material for: Highly Selenite-Tolerant Strain Proteus mirabilis QZB-2 Rapidly Reduces Selenite to Selenium Nanoparticles in the Cell Membrane
Source: Front Microbiol. 2022 Apr 11;13:862130. doi: 10.3389/fmicb.2022.862130 (PMC9037631; doi:10.3389/fmicb.2022.862130)
Supplement: Supplementary file 1 [file Table_1.doc]

>QZB-2

TCTGAAACTGGTTG-GCTAGAGTCTTGTAGAGGGGGG-TAGAATTCC-ATGTGTAGCGGTGAAATGCGTAGAGATGTGGAGGAATACCGGTGGCGAAGGCGGCCCCC-TGGACAAAGACTGACGCTCAGGTGCGAAAGCGTGGGGAGCAAACAGG-ATTAGATACCCTGG-TAGTCCACGCTGTAAACGATGTCGATTTAGAGGTTGTGGTCTTGAACCG-TGGCTTCTGGAGCTAACGCGTTAAATCGACCGCCTGGGGAGTACGGCCGCAAGGTTAAAACTCAAATGAATTGACGGGGGCCCGCACAAGCGGTGGAGCATGTGGTTTAATTCGATGCAACGCGAAGAACCTTACCTACTCTTGACATCCAGCGAATCCTTTAGAGATAGAGGAGTGCCTTCGGGAACGCTGA-GACAGGTGCTGCATGGCTGTCGTCAGCTCGTGTTGTGAAATGTTGGGTTAAGTCCCGCAACGAGCGCAACCCTTATCCTTTGTTGCCAGCACGTAATGGTGGGAACTCAAAGG-AGACTGCCGGTGATAAACCG-GAGGAAGGTGGGG-ATGACGTCAAGTCATCATGGCCCTTACGAGTAGGGCTACACACGTGCTACAATGGCAGATACAAAGAGAAGCGACCTCGCGAGAGCAAGCGGAACTCATAAAGTCTGTCGTAGTCCGGATTGGAGTCTGCAACTCGACTCCATGAAGTCGGAATCGCTAGTAATCGTAGATCAGAATGCTACGGTGAATACGTTCCCGGGCCTTGTACACACCGCCCGTCACACCATGGGAGTGGGTTGCAAAAGA
